# Untold Stories in User-Centered Design of Mobile Health: Practical Challenges and Strategies Learned From the Design and Evaluation of an App for Older Adults With Heart Failure

**DOI:** 10.2196/17703

**Published:** 2020-07-21

**Authors:** Victor Philip Cornet, Tammy Toscos, Davide Bolchini, Romisa Rohani Ghahari, Ryan Ahmed, Carly Daley, Michael J Mirro, Richard J Holden

**Affiliations:** 1 Department of Human-centered Computing School of Informatics and Computing IUPUI Indianapolis, IN United States; 2 Parkview Mirro Center for Research and Innovation Parkview Health Fort Wayne, IN United States; 3 Department of BioHealth Informatics School of Informatics and Computing IUPUI Indianapolis, IN United States; 4 Department of Medicine School of Medicine Indiana University Indianapolis, IN United States; 5 Regenstrief Institute Indianapolis, IN United States

**Keywords:** user-centered design, research methods, mobile health, digital health, mobile apps, usability, technology, evaluation, human-computer interaction, mobile phone

## Abstract

**Background:**

User-centered design (UCD) is a powerful framework for creating useful, easy-to-use, and satisfying mobile health (mHealth) apps. However, the literature seldom reports the practical challenges of implementing UCD, particularly in the field of mHealth.

**Objective:**

This study aims to characterize the practical challenges encountered and propose strategies when implementing UCD for mHealth.

**Methods:**

Our multidisciplinary team implemented a UCD process to design and evaluate a mobile app for older adults with heart failure. During and after this process, we documented the challenges the team encountered and the strategies they used or considered using to address those challenges.

**Results:**

We identified 12 challenges, 3 about UCD as a whole and 9 across the UCD stages of formative research, design, and evaluation. Challenges included the timing of stakeholder involvement, overcoming designers’ assumptions, adapting methods to end users, and managing heterogeneity among stakeholders. To address these challenges, practical recommendations are provided to UCD researchers and practitioners.

**Conclusions:**

UCD is a gold standard approach that is increasingly adopted for mHealth projects. Although UCD methods are well-described and easily accessible, practical challenges and strategies for implementing them are underreported. To improve the implementation of UCD for mHealth, we must tell and learn from these traditionally untold stories.

## Introduction

The user-centered design (UCD), or human-centered design, is a framework for iteratively researching, designing, and evaluating services and systems by involving end users and other stakeholders throughout a project life cycle. [1-3]. Mobile health (mHealth) projects benefit from UCD by using input from patients, informal caregivers, clinicians, and other stakeholders during the project life cycle to create better designs and iteratively improve interventions, thus enhancing their usability, acceptance, and potential success when implemented [4-6]. Increasingly, UCD has been recommended and adopted in mHealth projects to great success [7], with many examples of mHealth for people living with HIV [5,8], chronic conditions [9-11], or mental illness [6,12,13].

UCD is supported by popular tools and methods, such as cognitive task analysis, workflow studies and journey mapping, participatory design, rapid prototyping, usability testing, and heuristic evaluation [14-17]. Textbooks, articles, and other resources offer easily accessible and detailed guidance on the general UCD process and specific UCD methods [18,19]. However, an informal review of the literature reveals little information about the practical implementation of UCD methods.

Practical challenges reported in studies largely in non–health care domains include ensuring participants’ representativeness of the target population [20]; threats to innovation [21]; difficulty communicating with people from different backgrounds [22]; and organizational barriers, such as not having convenient access to participants [23].

Although studies applying UCD for mHealth are on the rise, very few mHealth studies report the challenges they face while planning and executing UCD activities, or they do so parenthetically. UCD challenges may be unique or amplified in the field of mHealth. For example, there are known difficulties in evaluating the effectiveness of mHealth solutions, in part because of the variable and multifactorial nature of health and illness trajectories [6]. mHealth projects often involve unique stakeholders, drawn from vulnerable patient populations (eg, older adults, patients with chronic conditions) [24,25] and busy clinician experts [6,26], or sometimes both. Moreover, on the one hand, there is sometimes a mismatch between the use of UCD methods (eg, rapid prototyping, user testing) and emerging technologies (eg, sensors) and, on the other hand, traditions (eg, clinical trials) and technological conservatism that characterize much of the health care sector [27-29].

Given the presence and importance of the practical challenges for implementing UCD, coupled with the increased use of UCD for mHealth, we argue for the need to explicitly describe those challenges. As mHealth technologies become more pervasive, navigating practical UCD challenges is essential for the development of “safe, sound, and desirable” [30] mHealth solutions that improve health outcomes while involving stakeholders in the design process [31,32]. We believe that identifying, reporting, and discussing the *untold stories* of actually implementing UCD for mHealth will help in overcoming the significant gaps between research and practice [33,34].

## Methods

### Overview

The objectives of this study were as follows:

- Characterize practical challenges encountered while implementing UCD to design an mHealth app for older adults with heart failure.
- Discuss strategies that we used or considered using to manage these challenges.

In presenting these challenges and strategies, we offer illustrations from our own experiences, particularly the Power to the Patient (P2P) project (R21 HS025232) and cite others that have been described in the literature in and outside the mHealth arena.

### Power to the Patient Project

From 2017 to 2019, we performed a UCD study to design and evaluate information technology for older adults with heart failure. On the basis of previous literature, we knew that these patients had unmet needs and required additional support to monitor and manage symptoms and various related behaviors, including medication use, dietary and fluid restriction, and physical activity [35-37]. Our work focused on delivering information and decision support by leveraging a novel technological opportunity, namely, sharing with patients the data from their cardiac implanted electronic devices (CIEDs). Many patients with heart failure have CIEDs for the delivery of timely cardiac therapy and the capturing of data that can predict decompensation and other events leading to hospitalization and other downstream outcomes [38,39]. Patients seldom receive their CIED data [40,41], but technical and cultural changes are increasing the likelihood that they will in the future [41-43].

**Figure 1:**
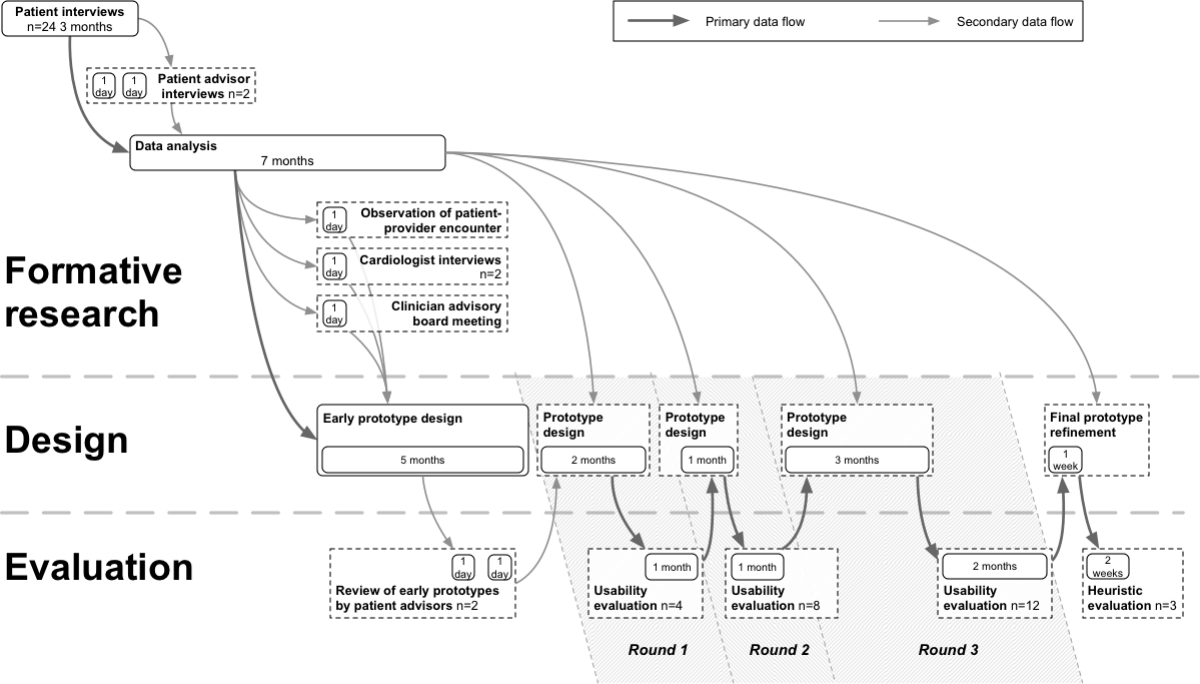
Timeline of the Power to the Patient project.

Figure 1 presents an overview of the project’s timeline, and Textboxes 1 and 2 further elaborate on the methods used. Select methods and results from the project are available elsewhere [19,32,44-47].

The project began with a *problem analysis* or formative study of the domain as a precursor to design. This phase comprised interviews with 24 older adults with heart failure, half of whom had CIEDs, to learn about how they made decisions about their health. These interviews used the critical incident technique, a method that asks participants to recall and describe a specific event or scenario and uses probes to better understand the participants’ thoughts and actions during the event or scenario [48,49]. We also examined participants’ decision-making strategies in response to fictitious scenarios [16]. In these scenarios, individuals were presented with hypothetical situations related to data from a fictitious CIED and asked to think aloud as they made decisions about how to respond. Other formative study methods were a brief observation of a device clinic, meetings with 2 cardiologists, and sharing of findings and design work from 2 recent similar studies on CIED data sharing [43,50].

**Formative research methods used to establish the Power to the Patient’s domain space.**

**Patient interviews**

- Method: 70-min (1) critical incident interviews and (2) scenario-based cognitive interviews to understand the decision-making process of older adults with heart failure
- Participants: 24 English-speaking older adults (≥65 years) diagnosed with heart failure (New York Heart Association Class II-IV) and 14 accompanying support persons (family and friends). Patients were receiving care at Parkview Health (Fort Wayne, Indiana)
- Procedure: (1) Participants were asked to describe a recent minor adverse health event and were probed with questions about their thought, feelings, and actions. (2) Participants were presented with a picture of a fictitious device that could give them a CIED score representing their heart health; they were asked to describe what they would think and do depending on the score displayed on the device

**Patient advisory meetings**

- Method: One-on-one meetings with patient advisors soliciting feedback on (1) personas and use-case scenarios and (2) early design concepts and prototypes
- Participants: 2 older adults with heart failure from the community (Indianapolis, Indiana) who voluntarily assisted the study in an advisory capacity
- Procedure: (1) Advisors met with the research team to discuss the preliminary findings from the interviews and early persona development. They provided feedback on the findings, methods, and relevance of the work. (2) Advisors were presented with design alternatives of a Power to the Patient prototype, and then they interacted with it while thinking aloud using a computer and a mouse

**Clinician advisory board meeting**

- Method: Group dinner with clinician experts to elicit feedback on personas, use-case scenarios, and early concepts
- Participants: 7 Parkview Health clinicians (2 cardiologists, a device clinic supervisor, 2 technicians, a nurse, and the vice president of operations for the Parkview Heart Institute)
- Procedure: Personas and scenarios were presented, among other findings, and questions were asked of clinicians regarding the validity of the findings and related current protocols (some of which were subsequently collected)

**Individual interviews with 2 cardiologists**

**Observation of clinical encounters with a patient in the device clinic**

**Evaluation methods used during the Power to the Patient development.**

**Usability evaluations, round 1 (R1) and round 2 (R2)**

- Method: 90-min task-driven evaluation sessions of Power to the Patient prototypes to assess usability (primarily) and acceptability (secondarily)
- Participants: 4 (R1) and 8 (R2) English-speaking older adults diagnosed with heart failure and 3 accompanying support persons (2 in R1 and 1 in R2)
- Procedure: Participants performed specific tasks in the prototype while thinking aloud. Testing occurred in a private room, with an interactive prototype made in Axure RP 9 running on a Samsung Galaxy S7 smartphone. Participants’ manual interactions were video recorded. Pretest, participants completed a demographic survey (ie, age, gender, technology use, and education level) and the Newest Vital Sign health literacy screening (NVS) [51]; posttest, they completed the system usability scale [52], National Aeronautics and Space Administration Task Load Index (NASA-TLX) [51], and user acceptance survey. Participants were also interviewed about their understanding and projected use of Power to the Patient prototypes.

**Usability evaluation, round 3 (R3)**

- Method: 90-min scenario-driven evaluation sessions of Power to the Patient prototypes to assess acceptability (primarily) and usability (secondarily)
- Participants: 12 English-speaking older adults diagnosed with heart failure, with cardiac implanted electronic devices, and accompanied by 5 support persons
- Procedure: Participants simulated days 1 and 10 of longitudinal use of the Power to the Patient prototype while thinking aloud. They completed the same assessments as in earlier rounds and were interviewed about their understanding and projected use of the Power to the Patient prototype.

**Heuristic evaluation**

- Method: Heuristic evaluation questionnaire to assess usability of Power to the Patient prototypes
- Participants: 3 user-centered design experts external to the team
- Procedure: Participants explored the prototype based on 2 use cases. They then reported their observations for 9 heuristics and gave an overall rating for the usability of the prototype

Findings from the formative study were analyzed to develop personas, representing distinct ways patients made decisions, and use-case scenarios, representing decision-making situations in which hypothetical patients with CIEDs might find themselves. A model was also created depicting the flow of naturalistic decision making for heart failure self-care. These products were presented separately to 2 patient advisors and a panel of clinicians, who provided feedback on the realism and relevance of the personas and scenarios. The personas, use-case scenarios, and a review of the literature and market landscape (eg, app store reviews of similar mHealth products) were used to formulate requirements and early design concepts to be presented to patient advisors.

The design involved writing requirements and 5 months of iterative prototyping, concluding with an interactive prototype. Subsequently, we performed 3 rounds of formal laboratory-based usability testing with 24 participants, interleaved with periods of prototype redesign. Each round had a more complete prototype and an increasing number of participants (ie, n=4 in round 1, n=8 in round 2, and n=12 in round 3). The project concluded with a final refinement of the prototype and formal *heuristic* evaluations by 3 outside UCD experts.

The P2P app designed in this study had 4 core patient-facing components: a heart health score calculated from CIED data (a fictitious concept inspired by existing research [53,54]), self-assessments on recommended heart failure self-care domains (eg, medication use, sodium-restricted diet), tips and strategies for better self-management, and logs of data captured by the app. We designed and tested different implementations of these core concepts, changing information architecture and amending feature sets as we received feedback from study participants. For example, in one iteration, assessments, tips, and strategies were organized using the concept of *plans* that users could select.

## Results

### Overview

**Figure 2:**
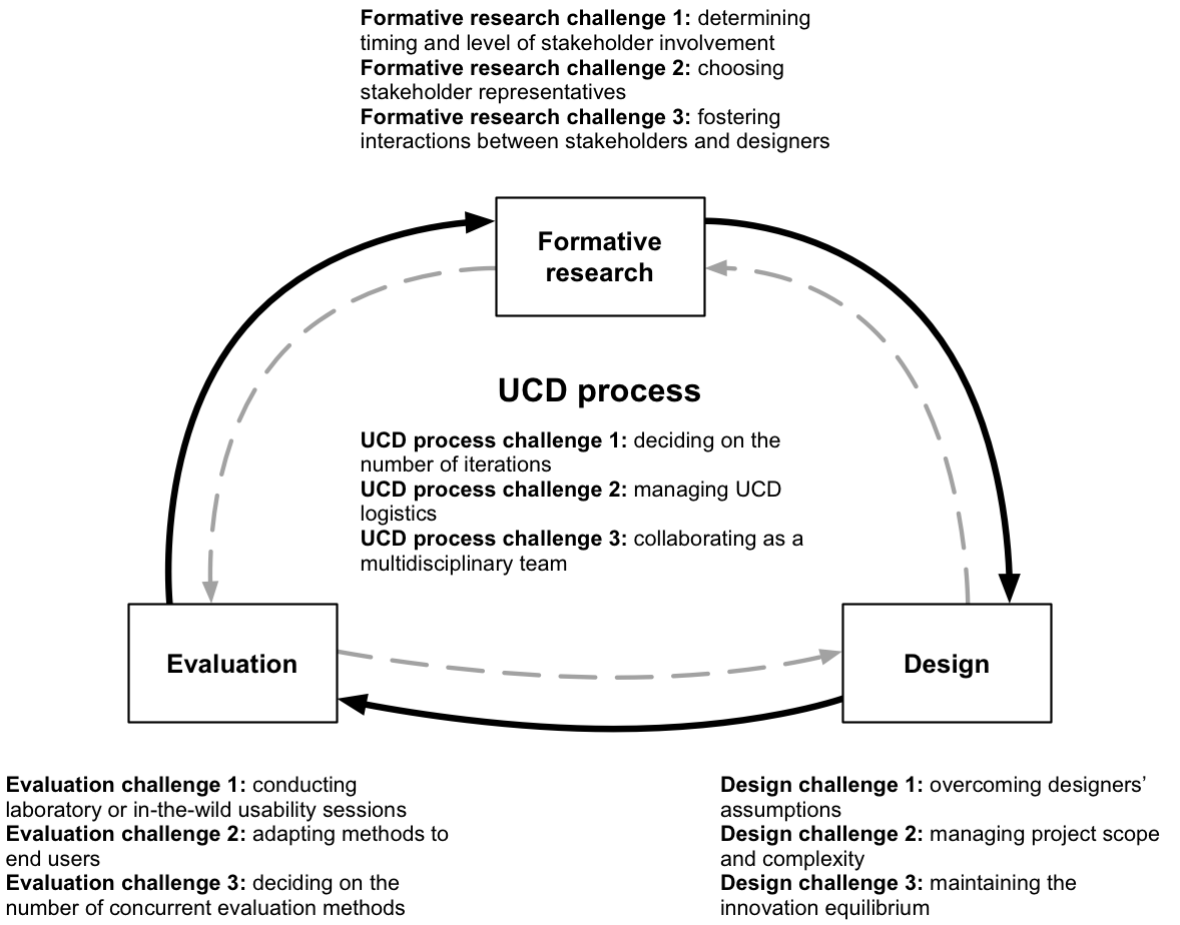
UCD challenges encountered during Power to the Patient research, development, and evaluation. UCD: User-centered design.

The UCD process as a whole and the 3 major UCD phases of formative research, design, and evaluation present associated implementation challenges. Twelve of the most pervasive challenges are summarized in Figure 2. Next, we discuss each, with examples from our experience with P2P, supplemented by relevant literature.

### Whole User-Centered Design Process

We identified 3 challenges related to the UCD process as a whole: deciding on the number of design-and-test iterations (UCD process challenge 1); managing logistics associated with UCD projects, such as preparing materials and recruitment costs (UCD process challenge 2); and collaborating as a multidisciplinary team by navigating misaligned goals and communication breakdowns (UCD process challenge 3). Recommendations for the UCD process are listed in Textbox 3.

**Recommendations for the user-centered design process.**

1. Keep the number of iterations flexible, estimating the range based on available resources but adjusting as the project progresses.
2. Avoid endless iterations by having a clear criteria for when to end the design and testing activities.
3. Consult or collaborate with experienced practitioners to anticipate and manage logistic challenges.
4. Explicitly discuss and seek to align multidisciplinary team members’ goals and preferences early on, managing these over time through open communication.
5. Involve a multilinguistic conductor to lead the team and coordinate members with diverse values, norms, practices, vocabularies, theories, and methods.

#### User-Centered Design Process Challenge 1: Deciding on the Number of Iterations

We planned the number of design-and-test iterations early in the project to satisfy the requirements of the funding agency and Institutional Review Board (IRB). Three iterations were originally estimated as feasible, given the timeline and amount of funding. Each iteration was assumed to require an average of at least 2 months of time and budget. As the project progressed, it became apparent that those assumptions were correct and that to adhere to budget and project timeline restrictions, we would be able to complete the 3 planned iterations. The number of participants in total and per iteration was based on several considerations. The total of 24 test participants were based on the project budget and timeline, as above, and the goal of having an average of 8 participants per test, a typical upper range for formative testing. For the initial 2 iterations, our focus was on identifying overt usability issues, and based on Nielsen’s recommendations, we planned to enroll about 4 to 8 participants (a few participants can uncover most usability issues [55]). In the last round, as our focus shifted toward evaluating acceptance and the extent to which the design was usable across a more diverse set of users, we recruited 12 participants.

Iterations in UCD benefit software development by reducing usability issues and improving features before it is too late or too expensive to make changes [56]. Iteration is generally recommended [56], but the number of iterations is not fixed and often depends on the project and how it progresses. However, deviating from a planned number of deviations can be costly or prohibited for other (eg, regulatory or management) reasons. Conversely, endless iteration is counterproductive and delays in-the-wild field testing and actual implementation.

#### UCD Process Challenge 2: Managing UCD Logistics

Many of our recurring challenges were related to logistics, such as meeting recruitment goals, preparing and managing materials (ie, instructions, presentations, and design documentation), and arranging a specific time for members and advisors to meet despite being geographically dispersed. Others have reported similar challenges in mHealth projects [57,58], but more often they are assumed and discussed informally between practitioners. This means that novice UCD practitioners tend to underestimate logistic challenges and have limited information on how to address them. UCD logistics challenges often mirror challenges to implementing patient-centered research in general, for example, the taxonomy of challenges by Holden et al [59], which includes patient identification and recruitment; privacy and confidentiality; conflicts with compensation; and logistical issues, such as travel, timing, and communication. Authors have described strategies to achieve buy-in, trust, transparency, accommodation (flexibility), openness, and anticipation [59,60], as well as checklists for implementing these general strategies [61].

Others have described the additional costs of recruiting end users for UCD projects [20,57]. It is even more difficult to recruit a representative sample in a technology project when technology ownership and proficiency are among the eligibility criteria [62] or the notion of technology leads individuals to decline participation [63]. Age- and illness-related physical and cognitive limitations may also exclude some individuals from mHealth studies [6,64]. In such cases, UCD projects must also consider whether to involve informal caregivers or others (eg, translators) who could assist patients during formative research or testing.

#### UCD Process Challenge 3: Collaborating as a Multidisciplinary Team

Our P2P project was the fruit of collaboration between a research university and a research center in a large health system, with support from patient and clinician advisors. Our team included a cardiologist coinvestigator who provided invaluable clinical information, feedback, and access to local and national clinical leaders. However, with disciplinary diversity comes disagreement, communication difficulty, and differences in assumptions, and although these are all desirable elements, they require efforts to manage, for example, by frequently asking team members to state their assumptions.

Multidisciplinary collaboration is often encouraged in UCD [6,65,66], including partnerships between designers and clinicians [67,68]. Those who have attempted such collaborations are aware of the methodological and cultural misalignment or divergent goals between Human-Computer Interaction technologists and clinicians [65,69]. Strategies to overcome these collaboration challenges include structured communication and the involvement of a *multilinguistic (symphonic) conductor*, a person who has learned “each team member’s discipline- or profession-specific values, norms, practices, vocabularies, theories, and methods to coordinate and translate between dissimilar members” [70].

### Formative Research

We encountered 3 types of formative research challenges in the P2P project: determining when and how to involve stakeholders (formative research challenge 1), recruiting participants and advisors who are representative of stakeholder groups (formative research challenge 2), and fostering meaningful interactions between stakeholders and designers despite personnel constraints (formative research challenge 3). Recommendations for formative research are listed in Textbox 4.

**Recommendations for formative research.**

1. Involve stakeholders early.
2. Avoid over recruiting or collecting more data than can be expediently analyzed.
3. Use shorter-cycle iterative research sprints with smaller sample sizes instead [55].
4. Carefully balance expectations placed on stakeholders versus what they are able or willing to do.
5. Deprioritize but do not discard the ideas that stakeholders rejected early on before the ideas reached maturity.
6. When possible, generate involvement from multiple stakeholder groups.
7. Use diverse recruitment methods to ensure stakeholders are chosen for representativeness, not convenience.
8. Foster direct relations between designers and stakeholders.
9. Minimize avoidable personnel changes and practice cross-staffing across user-centered design phases.

#### Formative Research Challenge 1: Determining Timing and Level of Stakeholder Involvement

We involved patients and clinicians early in the P2P project in several ways, including informant interviews and feedback on analyses, requirements, and early design concepts. This helped learn lessons early, such as clinicians insisting that because of interindividual variability in physical activity, activity goals should be highly individualized, whereas other goals (eg, medication adherence) could be identical for all users. Early learning allowed earlier decisions about scope, facilitated evidence-based design choices, and prevented having to make costly future design revisions. In terms of the scope, early stakeholder involvement helped eliminate especially difficult or risky design concepts, for example, including medication titration advice.

Early involvement was not always simple. The number and depth of P2P interviews yielded more data than could be analyzed in the time allotted. As a result, several important findings from stakeholder interviews were not discovered until further in the design process, negating some of the benefits of early learning. Furthermore, having sought feedback early may have prematurely terminated concepts that were promising but premature at the time.

The forms of involvement vary from collecting extensive data to asking individuals to assess early products [65]. The level of involvement can also be adjusted between informing (as in interviews), advising (as in reviewing concepts), and doing (as in having stakeholders co-perform research or design work) [71]. However, more active or laborious stakeholder involvement risks asking individuals to do more than what is realistic, reasonable, or affordable [21,72]. This is often the case when individuals are asked to be co-designers without adequate training in design, compensation for their contribution, or understanding of the problem space. Although some involvement is essential to UCD, more is not always better [71].

#### Formative Research Challenge 2: Choosing Stakeholder Representatives

P2P was fortunate to obtain input from patients sampled from a pool of current patients; volunteer advisors who were willing to meet repeatedly with the design team; and various clinicians, some of whom also offered access to their clinic and protocols. Not every design team can easily access stakeholders for formative research in a timely manner, far less multiple stakeholder groups, especially when the stakeholders include busy professionals. Some researchers resort to gathering data from less representative convenience samples, including online services offering access to paid volunteers, such as Amazon’s Mechanical Turk or the Qualtrics Panel [73-75].

Despite having adequate access, P2P was also limited in the variety and representativeness of stakeholders. The patients we interviewed in our formative research were all white, and two-third were male. Patient advisors were more likely to be educated and engaged in their health than peers, consistent with the general trend that patient advisors are rarely *ordinary people*. Clinicians in our study may have been more motivated than nonparticipants. Although stakeholder involvement is essential to UCD [1,76], it is predicated on stakeholders having unique knowledge or insights that designers do not have. However, stakeholders too have limited knowledge and represent primarily the communities to which they belong, meaning even with stakeholder involvement, there may exist multiple blind spots. When those who are involved differ from end users (eg, on race, education, or motivation), those blind spots may disadvantage underrepresented groups [63]. In practice, however, few design studies have the opportunity to conduct formative research with large samples representative of the population, whereas increasing sample size exacerbates formative research challenge 1 (“When and how much to involve stakeholders”), as discussed above.

Those who have worked extensively with patient advisory boards offer useful advice on assembling the right group of stakeholders, especially when they must work together, as on a panel. Suggestions include leadership commitment to listening to stakeholder suggestions, diverse recruitment (to avoid the abovementioned blindspots), careful selection of individuals who will work well with others, and adequate funding to compensate or otherwise support stakeholders [77].

#### Formative Research Challenge 3: Fostering Interactions Between Stakeholders and Designers

We attempted to promote direct interactions between designers and both patient and clinician stakeholders. Designers attended many of the formative research sessions or had direct access to the collected raw data. Furthermore, to ensure continuity, there was cross-staffing of formative research, design, and evaluation teams. One project member personally participated in almost every interview, feedback session, design meeting, and usability test. However, she was the only design team member who had interviewed patients and was therefore expected to be the *voice* of patient participants on the design team. Over time, turnover greatly reduced the number of team members who had been present from the beginning of the project and had therefore participated in any formative research activities.

Having designers interact directly with stakeholders, and especially end users, has been shown to yield better results [78] than hearing about the stakeholders and end users from another source [71]. Continual interaction with stakeholders during the UCD process helps designers gain a firsthand experience of the domain [78,79]. However, when projects progress sequentially from formative research with stakeholders to design and evaluation, turnover and staffing limitations may mean that those designing or testing the product may not have had such firsthand experience.

### Design

We identified 3 challenges related to the design phase of UCD: overcoming designers’ assumptions with empirical research findings (design challenge 1), managing project scope and complexity and avoiding *scope creep* (design challenge 2), and maintaining the *innovation equilibrium* by balancing new ideas with outside constraints (design challenge 3). Recommendations for design are listed in Textbox 5.

**Recommendations for design.**

1. Engage stakeholders during design as ad hoc informants or co-designers to challenge incorrect assumptions.
2. Conduct iterative new rounds of data collection during the design phase as questions arise that are best answered by gathering evidence.
3. Seek simplicity and thus reducing complexity.
4. Monitor for scope creep [80] and overly complex designs, relative to what end users need.
5. Plan for feature deimplementation (ie, removing features from design), using techniques such as a formal *termination plan* [33].
6. Without stifling innovation, ensure stakeholders can rule out designs that are unsafe, unacceptable, infeasible, inconsistent with clinical reality, or otherwise impractical.
7. For innovative ideas transcending conventional practice, develop clear plans for how the design will fit in or overcome existing infrastructure constraints, regulations, preferences, and habits.

#### Design Challenge 1: Overcoming Designers’ Assumptions

Designers naturally make decisions that are inspired by but immediately validated by end user evidence. Some of these decisions are based on assumptions that go unquestioned during design but are discovered to be incorrect during testing. This situation underscores the value of testing and the limitations of design. In the P2P project, for example, we incorporated rewards based on the literature. Nothing in the formative data contradicted the potential value of rewards, so it was not until testing that we learned that most participants thought the rewards were distracting. Some assumptions are also persistent and can be made despite disconfirming formative research findings. For example, members of the design team persistently believed that end users would have little technology experience, despite evidence to the contrary from formative research and usability testing.

Designer bias is difficult to overcome, even when UCD methods are used to collect contradictory evidence. The sequence of design following formative research means some assumptions are not tested or contradicted until the testing phase, by which time the assumptions may have greatly influenced the design. An alternative would be to conduct additional research to challenge the design team’s assumptions during the design phase, but before formal testing [81]*.* Furthermore, designers should be judicious in the use of design techniques, such as personas, which can lead to oversimplification and encourage misleading assumptions about end users [82]. Other strategies to mitigate incorrect assumptions include conducting more frequent testing or including stakeholders on design teams to challenge assumptions during the design process [50,71].

#### Design Challenge 2: Managing Project Scope and Complexity

Similar to other design projects, P2P produced many ideas, which were easier to generate than to dismiss. As a result, we attempted to include in a single app a large variety of features. We also attempted to integrate these many features to produce a coherent product. Often, multiple features were being slowly designed in parallel, rather than perfecting 1 feature before moving on to the next. These conditions sometimes led to confusion about the purpose of the app. More features also meant less time and effort spent designing or testing each.

Complexity and scope need to be carefully managed to avoid natural tendencies to add (rather than subtract) from taking over. Additional research could be used to help prioritize features and determine which features are attractive to designers but not needed by end users [28]. When a project’s scope is intentionally large, steps can be taken to create distinct modules (chunks of features) [83], which provide coherent structure and separation. If complexity is inevitable, the project team will need to plan for more extensive testing by conducting longer sessions or sessions with more users.

#### Design Challenge 3: Maintaining the Innovation Equilibrium

In our experience, designers, clinician stakeholders, and patient stakeholders were divided on what was possible for and needed from the product being designed. Generally, clinicians were more conservative, preferring to replicate existing practices and avoid less studied or riskier options. For instance, clinicians were more conservative than designers about how much unedited information and control over its interpretation to offer patients. Another point of contention was whether to integrate the product into other health information systems, including electronic medical records. Patients preferred integration, whereas designers were divided on leveraging those systems at the expense of their practical limitations and regulatory constraints. Innovation also conflicted with clinical reality, a case where a patient or designer might envision something that is not technically possible or clinically relevant [32]. For example, the design team assumed an ability to predict heart failure events through CIED data that were beyond publicly available scientific knowledge. Designers’ innovative ideas could also be mismatched with what patient end users were used to and could comfortably perform. This may have been the case with patients’ dislike of rewards or reluctance to rate their health using standard online rating conventions (eg, out of 5 stars). In general, end users tend to have more conventional preferences than designers [26]. In mHealth projects, patients may be unaware of or reluctant to suggest all the technological possibilities granted by smartphones [29], such as push notifications [84] or smartphone sensors [27].

In conversations with innovators, UCD professionals often hear the statement attributed to Henry Ford, “If I had asked people what they wanted, they would have said faster horses.” The broader challenge is maintaining the *innovation equilibrium*: allowing innovators to innovate, while also allowing stakeholders to influence or evaluate their design, especially when it comes to usability, safety, and privacy. The related challenge is to prevent innovation from creating products that commit what Cornet et al [32] call *type 2 design error*, which “occurs when designers do not accommodate the clinical reality, including biomedical knowledge, clinical workflows, and organizational requirements.”

### Evaluation

We identified 3 evaluation challenges: managing the tradeoff between laboratory and in-the-wild usability sessions (evaluation challenge 1); adapting standardized methods to the end user population, in our case, older adults (evaluation challenge 2); and deciding on the number of concurrent evaluation methods relative to the effort spent setting up sessions and analyzing data (evaluation challenge 3). Recommendations for evaluation are listed in Textbox 6.

**Recommendations for evaluation.**

1. Use a laboratory setup for usability testing to improve efficiency and effectiveness.
2. Begin testing in the laboratory but transition to in-the-wild testing as time and budget allow.
3. Adapt methods to end user needs, when necessary, even if this means deviating from the standard.
4. Allow for flexibility and experimentation, at times sacrificing standardization.
5. Control the number of concurrent evaluation methods; use efficiency, pacing, and workload management strategies if multiple methods are implemented.

#### Evaluation Challenge 1: Conducting Laboratory or In-the-Wild Usability Sessions

P2P usability testing was conducted in a laboratory setting, albeit in meeting rooms without built-in usability or simulation equipment (eg, a control room, multicamera recording, eye tracking). Although this setting was adequate in most cases, it was at times inconvenient. The laboratory setting was more challenging because it required participants to test prototypes in a time and place dissimilar from the intended context of use. Participants spent 30 min using a prototype technology meant to be used for weeks, months, and years. They were then asked to project how they would use the technology in practice. In the third round of testing, scenarios were used to simulate several days in the prolonged use of the product to help participants project future acceptance and use. However, the cross-sectional and laboratory-based design of our testing limited our confidence in our findings regarding acceptance and future use, relative to findings of usability (eg, observed errors or subjective usability ratings).

Evaluating mHealth prototypes in a laboratory setting offers ideal conditions for detecting product software usability issues, such as navigation or layout issues. Such evaluation however lacks external validity in reproducing the context of the use of the mHealth product [85] and therefore fails to assess most issues related to product hardware usability, operating system usability, acceptance, and longer term outcomes (eg, changes in behavior or health) [6]. Laboratory evaluation is appropriate to quickly iterate on designs and address usability issues before in-the-wild testing to avoid fielding a poorly designed product. However, in-the-wild testing is expensive and time-intensive and may not be possible in every project.

#### Evaluation Challenge 2: Adapting Methods to End Users

We adapted the standard methods in several ways, including accommodating older adult participants. For example, we administered a simplified version of the System Usability Scale (SUS) self-report measure, which we developed specifically for older adults [9,86,87]. We built flexibility for breaks during testing, especially given the use of diuretic medications by patients with heart failure. We also discovered challenges that we had not anticipated, for example, a participant having difficulty completing computer-based surveys because of vision and motor impairment.

In the context of mHealth projects, adapting standardized usability evaluation methods to end users is often necessary to accommodate patient abilities and limitations. For example, most standardized usability scales have technical or difficult words [86]; thus, many studies edit these measures, for example, by changing the “cumbersome” in the SUS to “awkward” [88]. Although standardized methods ensure scientific reproducibility, rigidity in the UCD process can undermine the goal of iteratively improving a product, which often requires flexibility and experimentation [65]. If, for example, researchers discover that some older participants have difficulty using a touchscreen device, it may be worth adapting the protocol to permit the use of a mouse or stylus in subsequent testing.

#### Evaluation Challenge 3: Deciding on the Number of Concurrent Evaluation Methods

Our testing involved participant consenting, lengthy pre- and posttest surveys, posttest interviews, and task-based usability testing with think-aloud. In addition, each testing session required a pretest room and equipment setup and posttest aggregation of data from audio, video, computerized, and written recordings. At times, the multiplicity of methods in a single session resulted in testing sessions being cut short. Moreover, the amount of data collected during testing affected the speed at which the team could analyze usability test findings and prepare the next design for another round of testing.

Using multiple concurrent evaluation methods improves triangulation and therefore mHealth usability [89]. However, each method adds burden and affects the timeline. Thus, those implementing UCD should pursue strategies to reduce inefficiency (eg, use of a dedicated testing room to reduce setup labor), ensure pacing (eg, blocking off staff time for testing and analysis), and reduce workload (eg, use of automated usability data collection or analysis) [90].

## Discussion

### Limitations

The challenges reported were based on our experience with a P2P project, supplemented by firsthand experience with multiple other mHealth projects and a review of the literature. However, the literature yielded few explicit depictions of challenges and less formal discussion of them. (This validated the goals of this paper.) Both our experiences and most of those described in the literature originated in academic environments, which have unique staffing, timing, and funding characteristics. The UCD implementation challenges and strategies encountered in the industry may be different, although an examination of gray literature (eg, blog posts and popular design books) shows some similarities. Finally, our recommendations are to be taken with caution, as they have not been formally validated across projects, project teams, or environments. The mHealth UCD community should actively debate these recommendations and produce new ones.

### Conclusions

UCD implementation for mHealth apps can lead to highly usable and acceptable patient-centered and clinically valid solutions. Implementation is challenging, as the 12 practical challenges in this paper easily illustrate. However, these challenges can be overcome, and our recommendations may help others apply UCD to mHealth or similar arenas. Telling and learning from the typically *untold stories* will result in more efficient, effective, and sustainable mHealth design efforts, effectively bridging the gap between the science and practice of UCD and mHealth implementation. We call on our fellow researchers, designers, and UCD experts to document and share their own challenges and strategies toward improving the implementation of UCD.

## Abbreviations

AHRQ: Agency for Healthcare Research and Quality
CIED: cardiac implanted electronic device
mHealth: mobile health
P2P: Power to the Patient
SUS: system usability scale
UCD: user-centered design

## References

[1] (2010). Ergonomics of human-System Interaction — Part 210: Human-Centred Design for Interactive Systems. International Organization for Standardization.

[2] Gould J, Lewis C (1985). Designing for usability: key principles and what designers think. Commun ACM.

[3] Norman D (2013). The Design of Everyday Things: Revised and Expanded Edition.

[4] McCurdie T, Taneva S, Casselman M, Yeung M, McDaniel C, Ho W, Cafazzo J (2012). mHealth consumer apps: the case for user-centered design. Biomed Instrum Technol.

[5] Schnall R, Rojas M, Bakken S, Brown W, Carballo-Dieguez A, Carry M, Gelaude D, Mosley JP, Travers J (2016). A user-centered model for designing consumer mobile health (mHealth) applications (apps). J Biomed Inform.

[6] Ben-Zeev D, Schueller SM, Begale M, Duffecy J, Kane JM, Mohr DC (2015). Strategies for mhealth research: lessons from 3 mobile intervention studies. Adm Policy Ment Health.

[7] Marcolino MS, Oliveira JA, D'Agostino M, Ribeiro AL, Alkmim MB, Novillo-Ortiz D (2018). The impact of mhealth interventions: systematic review of systematic reviews. JMIR Mhealth Uhealth.

[8] Wray T, Kahler C, Simpanen E, Operario D (2019). User-centered, interaction design research approaches to inform the development of health risk behavior intervention technologies. Internet Interv.

[9] Srinivas P, Cornet VP, Holden RJ (2017). Human factors analysis, design, and evaluation of engage, a consumer health IT application for geriatric heart failure self-care. Int J Hum Comput Interact.

[10] Giunti G, Mylonopoulou V, Romero OR (2018). More stamina, a gamified mhealth solution for persons with multiple sclerosis: research through design. JMIR Mhealth Uhealth.

[11] Morita PP, Yeung MS, Ferrone M, Taite AK, Madeley C, Lavigne AS, To T, Lougheed MD, Gupta S, Day AG, Cafazzo JA, Licskai C (2019). A patient-centered mobile health system that supports asthma self-management (breathe): design, development, and utilization. JMIR Mhealth Uhealth.

[12] Vilardaga R, Rizo J, Zeng E, Kientz J, Ries R, Otis C, Hernandez K (2018). User-centered design of learn to quit, a smoking cessation smartphone app for people with serious mental illness. JMIR Serious Games.

[13] Biagianti B, Hidalgo-Mazzei D, Meyer N (2017). Developing digital interventions for people living with serious mental illness: perspectives from three mhealth studies. Evid Based Ment Health.

[14] Nielsen J (1994). Usability Engineering.

[15] Rubin J, Chisnell D (2008). Handbook of Usability Testing: How to Plan, Design, and Conduct Effective Tests.

[16] Crandall B, Klein GA, Klein G, Hoffman RR (2006). Working Minds: A Practitioner's Guide to Cognitive Task Analysis.

[17] Simonsen J, Robertson T (2012). Routledge International Handbook of Participatory Design.

[18] Holden RJ, Voida S, Savoy A, Jones JF, Kulanthaivel A, Finnel JT, Dixon BE (2016). Human factors engineering and human–computer interaction: supporting user performance and experience. Clinical Informatics Study Guide: Text and Review.

[19] Cornet VP, Daley C, Cavalcanti LH, Parulekar A, Holden RJ, Sethumadhavan A, Sasangohar F (2020). Design for self care. Design for Health: Applications of Human Factors.

[20] Kujala S, Kauppinen M (2004). Identifying and Selecting Users for User-Centered Design. Proceedings of the third Nordic Conference on Human-Computer Interaction.

[21] Heinbokel T, Sonnentag S, Frese M, Stolte W, Brodbeck F (1996). Don't underestimate the problems of user centredness in software development projects - there are many!. Behav Inf Technol.

[22] Shah LM, Yang WE, Demo RC, Lee MA, Weng D, Shan R, Wongvibulsin S, Spaulding EM, Marvel FA, Martin SS (2019). Technical guidance for clinicians interested in partnering with engineers in mobile health development and evaluation. JMIR Mhealth Uhealth.

[23] Matthew-Maich N, Harris L, Ploeg J, Markle-Reid M, Valaitis R, Ibrahim S, Gafni A, Isaacs S (2016). Designing, implementing, and evaluating mobile health technologies for managing chronic conditions in older adults: a scoping review. JMIR Mhealth Uhealth.

[24] Arsand E, Demiris G (2008). User-centered methods for designing patient-centric self-help tools. Inform Health Soc Care.

[25] Kushniruk A, Nøhr C (2019). Participatory design, user involvement and health IT evaluation. Stud Health Technol Inform.

[26] van Velsen L, Wentzel J, van Gemert-Pijnen JE (2013). Designing eHealth that matters via a multidisciplinary requirements development approach. JMIR Res Protoc.

[27] Cornet VP, Holden RJ (2018). Systematic review of smartphone-based passive sensing for health and wellbeing. J Biomed Inform.

[28] Mann DM, Quintiliani LM, Reddy S, Kitos NR, Weng M (2014). Dietary approaches to stop hypertension: lessons learned from a case study on the development of an mHealth behavior change system. JMIR Mhealth Uhealth.

[29] Fiordelli M, Diviani N, Schulz PJ (2013). Mapping mHealth research: a decade of evolution. J Med Internet Res.

[30] Albrecht U, von Jan U (2017). Safe, sound and desirable: development of mHealth apps under the stress of rapid life cycles. Mhealth.

[31] Becker S, Miron-Shatz T, Schumacher N, Krocza J, Diamantidis C, Albrecht U (2014). mHealth 2.0: experiences, possibilities, and perspectives. JMIR Mhealth Uhealth.

[32] Cornet VP, Daley C, Bolchini D, Toscos T, Mirro MJ, Holden RJ (2019). Patient-centered design grounded in user and clinical realities: towards valid digital health. Proc Int Symp Hum Fact Erg Health Care.

[33] Boustani MA, van der Marck MA, Adams N, Azar JM, Holden RJ, Vollmar HC, Wang S, Williams C, Alder C, Suarez S, Khan B, Zarzaur B, Fowler NR, Overley A, Solid CA, Gatmaitan A (2019). Developing the agile implementation playbook for integrating evidence-based health care services into clinical practice. Acad Med.

[34] Waterson P, Shorrock S, Williams C (2017). Ergonomics and ergonomists: lessons for human factors and ergonomics practice from the past and present. Human Factors and Ergonomics in Practice: Improving System Performance and Human Well-Being in the Real World.

[35] Roger VL (2013). Epidemiology of heart failure. Circ Res.

[36] Lainscak M, Blue L, Clark A, Dahlström U, Dickstein K, Ekman I, McDonagh T, McMurray JJ, Ryder M, Stewart S, Strömberg A, Jaarsma T (2011). Self-care management of heart failure: practical recommendations from the patient care committee of the heart failure association of the European society of cardiology. Eur J Heart Fail.

[37] Holden RJ, Schubert CC, Mickelson RS (2015). The patient work system: an analysis of self-care performance barriers among elderly heart failure patients and their informal caregivers. Appl Ergon.

[38] Klersy C, Boriani G, de Silvestri A, Mairesse G, Braunschweig F, Scotti V, Balduini A, Cowie MR, Leyva F, Health Economics Committee of the European Heart Rhythm Association (2016). Effect of telemonitoring of cardiac implantable electronic devices on healthcare utilization: a meta-analysis of randomized controlled trials in patients with heart failure. Eur J Heart Fail.

[39] Hawkins NM, Virani SA, Sperrin M, Buchan IE, McMurray JJ, Krahn AD (2016). Predicting heart failure decompensation using cardiac implantable electronic devices: a review of practices and challenges. Eur J Heart Fail.

[40] Mirro M, Daley C, Wagner S, Ghahari RR, Drouin M, Toscos T (2018). Delivering remote monitoring data to patients with implantable cardioverter-defibrillators: does medium matter?. Pacing Clin Electrophysiol.

[41] Daley CN, Allmandinger A, Heral L, Toscos T, Plant R, Mirro M (2015). Engagement of ICD patients: direct electronic messaging of remote monitoring data via a personal health record. EP Lab Digest.

[42] Daley CN, Chen EM, Roebuck AE, Ghahari RR, Sami AF, Skaggs CG, Carpenter M, Mirro M, Toscos T (2017). Providing patients with implantable cardiac device data through a personal health record: a qualitative study. Appl Clin Inform.

[43] Rohani Ghahari R, Holden RJ, Flanagan M, Wagner S, Martin E, Ahmed R, Daley CN, Tambe R, Chen E, Allmandinger T, Mirro MJ, Toscos T (2018). Using cardiac implantable electronic device data to facilitate health decision making: a design study. Int J Ind Ergon.

[44] Daley CN, Bolchini D, Varrier A, Rao K, Joshi P, Blackburn J, Toscos T, Mirro MJ, Wagner S, Martin E, Miller A, Holden RJ (2018). Naturalistic decision making by older adults with chronic heart failure: an exploratory study using the critical incident technique. Proc Hum Factors Ergon Soc Annu Meet.

[45] Daley CN, Cornet VP, Patekar G, Kosarabe S, Bolchini D, Toscos T, Mirro M, Wagner S, Martin E, Rohani Ghahari R, Ahmed R, Miller A, Holden RJ (2019). Uncertainty management among older adults with heart failure: responses to receiving implanted device data using a fictitious scenario interview method. Proc Int Sym Hum Fact Erg Health Care.

[46] Holden RJ, Joshi P, Rao K, Varrier A, Daley CN, Bolchini D, Blackburn J, Toscos T, Wagner S, Martin E, Miller A, Mirro MJ (2018). Modeling personas for older adults with heart failure. Proc Hum Factors Ergon Soc Annu Meet.

[47] Holden RJ, Daley CN, Mickelson RS, Bolchini D, Toscos T, Cornet VP, Miller A, Mirro MJ (2020). Patient decision-making personas: an application of a patient-centered cognitive task analysis (P-CTA). Appl Ergon.

[48] Kain D, de Marrais K, Lapan SD (2004). Owning significance: the critical incident technique in research. Foundations for Research: Methods of Inquiry in Education and the Social Sciences.

[49] Flanagan JC (1954). The critical incident technique. Psychol Bull.

[50] Ahmed R, Toscos T, Ghahari RR, Holden RJ, Martin E, Wagner S, Daley CN, Coupe A, Mirro MJ (2019). Visualization of cardiac implantable electronic device data for older adults using participatory design. Appl Clin Inform.

[51] Hart S, Staveland L (1988). Development of NASA-TLX (task load index): results of empirical and theoretical research. Adv Psychol.

[52] Brooke J, Jordan PW, Thomas B, McClelland IL, Weerdmeester B (1996). SUS: a 'quick and dirty' usability scale. Usability Evaluation In Industry.

[53] Boehmer JP, Hariharan R, Devecchi FG, Smith AL, Molon G, Capucci A, An Q, Averina V, Stolen CM, Thakur PH, Thompson JA, Wariar R, Zhang Y, Singh JP (2017). A multisensor algorithm predicts heart failure events in patients with implanted devices: results from the multisense study. JACC Heart Fail.

[54] Gardner RS, Singh JP, Stancak B, Nair DG, Cao M, Schulze C, Thakur PH, An Q, Wehrenberg S, Hammill EF, Zhang Y, Boehmer JP (2018). Heartlogic multisensor algorithm identifies patients during periods of significantly increased risk of heart failure events: results from the multisense study. Circ Heart Fail.

[55] Nielsen J, Landauer TK (1993). A Mathematical Model of the Finding of Usability Problems. Proceedings of the INTERACT '93 and CHI '93 Conference on Human Factors in Computing Systems.

[56] Nielsen J (1993). Iterative user-interface design. Computer.

[57] Baez M, Casati F (2018). Agile Development for Vulnerable Populations: Lessons Learned and Recommendations. Proceedings of the 40th International Conference on Software Engineering: Software Engineering in Society.

[58] Holden RJ, Bodke K, Tambe R, Comer RS, Clark DO, Boustani M (2016). Rapid translational field research approach for eHealth R&D. Proc Int Sym Hum Fact Erg Health Care.

[59] Holden RJ, Scott AM, Hoonakker PLT, Hundt AS, Carayon P (2015). Data collection challenges in community settings: insights from two field studies of patients with chronic disease. Qual Life Res.

[60] Holden RJ, Or CK, Alper SJ, Joy Rivera A, Karsh BT (2008). A change management framework for macroergonomic field research. Appl Ergon.

[61] Valdez RS, Holden RJ (2016). Health care human factors/ergonomics fieldwork in home and community settings. Ergon Des.

[62] Toscos T, Drouin M, Pater J, Flanagan M, Pfafman R, Mirro MJ (2019). Selection biases in technology-based intervention research: patients' technology use relates to both demographic and health-related inequities. J Am Med Inform Assoc.

[63] Holden RJ, Toscos T, Daley CN, Roscoe R, Chiou EK, Wooldridge AR (2019). Researcher reflections on human factors and health equity. Advancing Diversity, Inclusion, and Social Justice Through Human Systems Engineering.

[64] Cosco TD, Firth J, Vahia I, Sixsmith A, Torous J (2019). Mobilizing mHealth data collection in older adults: challenges and opportunities. JMIR Aging.

[65] Pagliari C (2007). Design and evaluation in ehealth: challenges and implications for an interdisciplinary field. J Med Internet Res.

[66] Poole ES (2013). HCI and mobile health interventions: how human-computer interaction can contribute to successful mobile health interventions. Transl Behav Med.

[67] Aidemark J, Askenäs L, Nygårdh A, Strömberg A (2015). User involvement in the co-design of self-care support systems for heart failure patients. Procedia Comput Sci.

[68] Blandford A, Gibbs J, Newhouse N, Perski O, Singh A, Murray E (2018). Seven lessons for interdisciplinary research on interactive digital health interventions. Digit Health.

[69] Calvo RA, Dinakar K, Picard R, Christensen H, Torous J (2018). Toward impactful collaborations on computing and mental health. J Med Internet Res.

[70] Holden RJ, Binkheder S, Patel J, Viernes SH (2018). Best practices for health informatician involvement in interprofessional health care teams. Appl Clin Inform.

[71] Kujala S (2003). User involvement: a review of the benefits and challenges. Behav Inf Technol.

[72] Baek EO, Cagiltay K, Boling E, Frick T, Spector JM, Merrill MD, van Merrienboer J, Driscoll MP (2008). User-centered design and development. Handbook of Research on Educational Communications and Technology: A Project of the Association for Educational Communications and Technology.

[73] Kittur A, Chi EH, Suh BW (2008). Crowdsourcing user studies with mechanical turk. Proceedings of the SIGCHI Conference on Human Factors in Computing Systems.

[74] Guillory J, Kim A, Murphy J, Bradfield B, Nonnemaker J, Hsieh Y (2016). Comparing Twitter and online panels for survey recruitment of e-cigarette users and smokers. J Med Internet Res.

[75] Walter SL, Seibert SE, Goering D, O’Boyle EH (2018). A tale of two sample sources: do results from online panel data and conventional data converge?. J Bus Psychol.

[76] Eshet E, Bouwman H (2016). Context of use: the final frontier in the practice of user-centered design?. Interact Comput.

[77] Sharma A, Angel L, Bui Q (2015). Patient advisory councils: giving patients a seat at the table. Fam Pract Manag.

[78] Keil M, Carmel E (1995). Customer-developer links in software development. Commun ACM.

[79] Béguin P (2003). Design as a mutual learning process between users and designers. Interact Comput.

[80] Lorenzi NM, Riley RT (2000). Managing change: an overview. J Am Med Inform Assoc.

[81] Magrabi F, Ong M, Coiera E (2016). Health IT for patient safety and improving the safety of health IT. Stud Health Technol Inform.

[82] Turner P, Turner S (2011). Is stereotyping inevitable when designing with personas?. Design Stud.

[83] Norman D (2011). Living with Complexity.

[84] Ahmed I, Ahmad NS, Ali S, Ali S, George A, Danish HS, Uppal E, Soo J, Mobasheri MH, King D, Cox B, Darzi A (2018). Medication adherence apps: review and content analysis. JMIR Mhealth Uhealth.

[85] Cho H, Yen PY, Dowding D, Merrill JA, Schnall R (2018). A multi-level usability evaluation of mobile health applications: a case study. J Biomed Inform.

[86] Cornet VP, Daley CN, Srinivas P, Holden RJ (2017). User-centered evaluations with older adults: testing the usability of a mobile health system for heart failure self-management. Proc Hum Factors Ergon Soc Annu Meet.

[87] Holden RJ, Campbell NL, Abebe E, Clark DO, Ferguson D, Bodke K, Boustani MA, Callahan CM, Brain Health Patient Safety Laboratory (2020). Usability and feasibility of consumer-facing technology to reduce unsafe medication use by older adults. Res Social Adm Pharm.

[88] Bangor A, Kortum P, Miller J (2008). An empirical evaluation of the system usability scale. Int J Hum-Comput Interact.

[89] Georgsson M, Staggers N (2016). An evaluation of patients' experienced usability of a diabetes mHealth system using a multi-method approach. J Biomed Inform.

[90] Maramba I, Chatterjee A, Newman C (2019). Methods of usability testing in the development of eHealth applications: a scoping review. Int J Med Inform.

